# Managing Alpha‐Gal Allergy in a Case of Bioprosthetic TAVR

**DOI:** 10.1155/crii/8190714

**Published:** 2026-04-24

**Authors:** Ruchi Patel, Aarav Patel, Ruchi Biswas, Benjamin Hsieh, Dhanasekaran Ramasamy, Chirag Patel

**Affiliations:** ^1^ Department of Medicine, New Jersey Medical School, Rutgers University, Newark, New Jersey, USA, rutgers.edu; ^2^ Somerset County Vocational and Technical High School, Bridgewater, New Jersey, USA; ^3^ Private Practice, Allied Digestive Health, Union, New Jersey, USA; ^4^ Private Practice, Ocean Allergy, Brick, New Jersey, USA

**Keywords:** alpha-gal syndrome, anaphylaxis, bioprosthetic valve, hypersensitivity, omalizumab, TAVR

## Abstract

Alpha‐gal syndrome (AGS) is an uncommon IgE‐mediated allergy to mammalian‐derived products, caused by sensitization to the oligosaccharide galactose‐α‐1,3‐galactose (alpha‐gal) after tick exposure. Although AGS is best recognized for delayed anaphylaxis following ingestion of red meat, its implications for exposure to mammalian‐derived biomaterials remain poorly characterized. We present an elderly woman with AGS who required transcatheter aortic valve replacement (TAVR) using a bovine bioprosthesis. An 84‐year‐old woman with serologically confirmed AGS following a Lone Star tick bite in 2012 presented with symptomatic severe aortic stenosis. Her clinical history included multiple allergic reactions to beef, persistently elevated IgE to beef, pork, and lamb, and a specific alpha‐gal IgE >100 kU/L. Following a multidisciplinary evaluation, she was not considered a candidate for a mechanical valve. A prophylactic regimen was implemented, including cetirizine, omalizumab, and 60 mg prednisone 6 h prior to TAVR. The procedure was successfully performed with a bovine‐derived bioprosthetic valve. Perioperative tryptase levels remained within normal range (5.2 µg/L), and no hypersensitivity reactions occurred. Postoperatively, prednisone was tapered, while fexofenadine and omalizumab were continued for 6 months. This case highlights that bioprosthetic valve replacement can be safely performed in AGS patients through multidisciplinary planning and individualized prophylaxis strategies. Further data are needed to develop standardized perioperative management protocols for this rare but clinically significant condition.

## 1. Introduction

Alpha‐gal syndrome (AGS) is a delayed IgE‐mediated hypersensitivity reaction directed against galactose‐α‐1,3‐galactose, a carbohydrate epitope expressed on non‐primate mammalian glycoproteins and glycolipids [1]. The condition is increasingly recognized in regions endemic to the *Amblyomma americanum* (Lone Star) tick, which mediates sensitization through cutaneous exposure to alpha‐gal in tick saliva [2]. Since its first description in 2009, AGS has become a well‐documented cause of delayed anaphylaxis after ingestion of red meat, typically occurring 3–6 h postexposure. Of note, hypersensitivity reactions may occur more rapidly depending on the route of allergen administration, as seen with intravenous exposure to alpha‐gal‐containing drugs, such as cetuximab [3]. This is especially relevant when considering alpha‐gal exposure in the perioperative setting. Beyond food‐related reactions, alpha‐gal sensitivity has been implicated in allergic responses to pharmaceutical and surgical materials containing mammalian products, including bioprosthetic cardiac valves [4, 5]. Patients requiring valve replacement present a unique challenge because most transcatheter and surgical bioprosthetic valves are derived from porcine or bovine tissue, both of which express alpha‐gal epitopes [6]. Furthermore, direct implantation of this allergen, analogous to intravenous exposure, raises concerns regarding perioperative reaction nature, timing, and severity [3]. Although several reports suggest that some AGS patients tolerate such implants without reaction, the absence of standardized management strategies poses a potential risk for perioperative anaphylaxis [7, 8]. Here, we describe the successful management of an elderly patient with serologically confirmed AGS undergoing transcatheter aortic valve replacement (TAVR) with a bovine bioprosthesis. This case underscores the importance of pre‐procedural risk mitigation, interdisciplinary coordination, and the potential role of biologic therapy in minimizing hypersensitivity risk.

## 2. Case Presentation

An 84‐year‐old woman with symptomatic severe aortic stenosis was referred for TAVR evaluation. Her medical history included hypertension, diabetes mellitus, coronary artery disease, chronic idiopathic urticaria, and confirmed AGS following a Lone Star tick bite in 2012. Serology demonstrated persistently elevated IgE to beef, pork, and lamb, with the most recent alpha‐gal specific IgE (sIgE) 65.80 kU/L, as seen in Table 1. Specific alpha‐gal IgE had previously been over 100 kU/L. She had a history of multiple reactions to beef, including one anaphylactic episode requiring hospitalization, but continued to tolerate dairy products.

**Table 1:** Patient’s most recent IgE levels.

Test	Result	Reference
Immunoglobulin E, total	436 IU/mL	6–495 IU/mL
F026‐IgE pork	30.70 kU/L	Class V (high)
F027‐IgE beef	50.60 kU/L	Class V (high)
F088‐IgE lamb	40.90 kU/L	Class V (high)
O215‐IgE alpha‐gal	65.80 kU/L	High

After careful consideration of the risks and benefits in the context of her alpha‐gal allergy, the heart team determined that a bioprosthetic valve remained the preferred choice over a mechanical valve. A multidisciplinary team comprising cardiology, allergy/immunology, anesthesiology, and infectious disease specialists developed a prophylactic plan. The regimen included cetirizine 20 mg daily initiated prior to procedure, omalizumab 300 mg initiated prior to procedure per allergist recommendation, and a single 60 mg prednisone dose 6 h before the procedure. In July 2025, she underwent a successful TAVR using a bovine‐derived bioprosthetic valve. Heparin and low molecular weight heparin were avoided, as heparin is mammalian‐derived and may contain alpha‐gal [6]. No intraoperative or postoperative hypersensitivity was observed. Perioperative tryptase remained within the normal range (5.2 µg/L). Prednisone was tapered postoperatively. On outpatient allergy follow‐up, omalizumab was continued for the patient’s urticaria as well as food allergy. Approval for omalizumab was obtained for food allergies. Fexofenadine 60 mg twice a day was also restarted for chronic urticaria. The patient remained free of new allergic or cardiovascular symptoms on follow‐up.

## 3. Discussion

AGS introduces complex considerations for patients undergoing invasive procedures involving mammalian‐derived biomaterials. The alpha‐gal epitope is expressed on porcine and bovine tissues, and IgE‐sensitized patients may theoretically experience immediate or delayed hypersensitivity to such materials [4]. However, available evidence suggests a wide spectrum of reactivity: while certain AGS patients experience reactions to gelatin or cetuximab, others tolerate bioprosthetic valves and bovine pericardial grafts without incident [7–10].

Importantly, Aykut et al. [7] demonstrated that patients with documented alpha‐gal sensitization who underwent bioprosthetic valve replacement experienced no perioperative allergic or anaphylactic reactions. These findings contrast with concerns that were largely derived by isolated case reports and retrospective analyses including a small number of patients, such as by Hawkins et al. [11]. At the same time, it is impossible to determine whether an adverse reaction would have occurred in our presented case in the absence of pre‐treatment. While the findings of Aykut et al. [7] raise reasonable doubt regarding the necessity of prophylaxis in sensitized patients, the alpha‐gal‐sIgE levels in their cohort were relatively modest. Our patient had markedly elevated alpha‐gal IgE levels, a degree of sensitization that may plausibly confer a higher risk. Hypersensitivity reactions to porcine‐derived heparin have been documented in AGS patients during cardiac surgery, suggesting that mammalian‐derived surgical materials may pose perioperative risks [11]. Subsequent studies have documented safe implantation when pretreatment protocols were used, such as perioperative corticosteroids, antihistamines, and, in some cases, omalizumab [12]. Omalizumab, an anti‐IgE monoclonal antibody, has shown benefit in preventing recurrent allergic episodes in AGS and other food allergies by reducing free IgE and downregulating mast‐cell activation [13]. Our case aligns with emerging evidence that careful perioperative planning, including preoperative IgE and sIgE quantification, a premedication regimen, and allergist consultation, can substantially reduce risk. The normal perioperative tryptase levels observed here further support the effectiveness of prophylaxis in preventing subclinical mast‐cell activation. Despite the literature outlining low prevalence adverse events in sensitized individuals, thoughtful risk stratification is still principal in high‐risk individuals. Given the aging population and increasing TAVR utilization, awareness of AGS in the structural heart population is critical. Clinicians should maintain a high index of suspicion for AGS in patients with histories of tick bites and delayed red‐meat allergy. Preprocedural screening, allergist involvement, and consideration of non‐mammalian alternatives (e.g., mechanical valves or polymeric options when feasible) should be part of shared decision‐making. Additional registry data and systematic reviews are needed to refine perioperative management algorithms and establish evidence‐based guidelines for AGS patients undergoing bioprosthetic implantation.

## Funding

This work did not receive any specific grant from funding agencies in the public, commercial, or not‐for‐profit sector.

## Consent

No written consent has been obtained from the patients, as there is no patient‐identifiable data included in this case report/series.

## Conflicts of Interest

The authors declare no conflicts of interest.

## Data Availability

Data sharing is not applicable to this article as no datasets were generated or analyzed during the current study.
